# Air Target ISAR Recognition Based on Data Augmentation and Transfer Learning

**DOI:** 10.3390/s26113323

**Published:** 2026-05-23

**Authors:** Moqian Wang, Zuzhen Huang, Jinjian Cai, Tao Wu, Youquan Lin

**Affiliations:** Nanjing Research Institute of Electronics Technology, Nanjing 210039, China

**Keywords:** air target, ISAR, prior information-guided, data augmentation, transfer learning

## Abstract

Aiming at the problems of extremely scarce measured samples and significant domain shift between simulated and measured data in automatic target recognition (ATR) of air targets for spaceborne radar, this paper proposes an inverse synthetic aperture radar (ISAR) image recognition method for air targets combining physics-driven data augmentation guided by detection prior information with domain adversarial transfer learning. First, the mapping relationship between scattering point projection and ISAR images is established by using the target 3D point cloud and radar observation geometric priors, and a 2D sinc kernel function is introduced for energy distribution rendering. Then, under the unsupervised transfer learning paradigm, aiming at the distribution inconsistency between augmented data (source domain) and unlabeled simulated data (target domain), this paper designs a cross-domain recognition task experiment including six types of typical aircraft targets, and compares the cross-domain recognition performance of three transfer learning methods (model fine-tuning, deep domain confusion (DDC) and domain-adversarial neural networks (DANN)) on the target domain. Meanwhile, t-distributed stochastic neighbor embedding (t-SNE) visualization is used to analyze the feature distribution alignment ability of the models. Simulation experiments show that the DANN model with a dynamic inversion coefficient introduced in the gradient reversal layer (GRL) achieves a recognition accuracy of 99.5% on the unlabeled target domain, which is significantly superior to the model fine-tuning and DDC methods. Moreover, it makes the feature distributions of source and target domain samples highly overlapping, and maintains a strong inter-class discriminability while eliminating the domain shift. The proposed scheme provides a physically interpretable and robust technical path for few-shot radar target image recognition.

## 1. Introduction

Spaceborne radar has become a core equipment in the integrated air and space defense system due to its advantages of wide-area coverage, all-weather operation and anti-stealth detection [1]. Realizing high-resolution imaging and automatic target recognition of non-cooperative air targets is the key to improving strategic early warning capabilities [2]. Inverse synthetic aperture radar (ISAR) can generate 2D high-resolution images through the relative motion of the target with respect to the radar, providing important feature support for target discrimination [3]. However, the high acquisition cost of measured ISAR images leads to a serious lack of samples for deep learning models; meanwhile, there is a significant data distribution difference between simulated and measured images, namely the domain shift problem, which severely restricts the generalization ability of recognition models [4].

Data augmentation is an effective means to alleviate sample scarcity [5]. Its essence is to generate the value equivalent to a larger amount of data on the basis of existing limited data, thereby improving the generalization ability of the model. Geometric transformation, color adjustment and other methods can effectively increase data diversity and improve the generalization and robustness of the model [6,7]. However, although traditional geometric transformation methods can expand the number of samples, it is difficult to simulate the physical characteristics of radar electromagnetic scattering varying with attitudes [8]. The emergence of generative adversarial networks (GANs) provides a new idea for data generation [9], but their training is difficult with a small number of samples and the quality of generated images is unstable [10,11].

In recent years, physics-driven data augmentation methods based on physical mechanisms have attracted attention. The attributed scattering center model can accurately characterize the electromagnetic scattering characteristics of targets, and decompose the target echo into the sum of the responses of several scattering centers [12,13]. The orthogonal matching pursuit algorithm can extract the position, amplitude and other parameters of scattering centers from SAR images [14]. Feng et al. [4] combined scattering features with GANs, constructed multi-dimensional feature inputs and introduced scattering similarity loss, so that the generated data maintains real scattering characteristics under both standard operating conditions and extended operating conditions [15]. Target recognition in SAR images can be realized by matching attributed scattering centers [16]. Such methods embed the laws of electromagnetic scattering into the generation process, providing a new path for improving sample quality [17].

In solving the domain shift problem, transfer learning, especially unsupervised domain adaptation technology, has shown great potential [18]. Explicit alignment of source and target domain features can be achieved by introducing the maximum mean discrepancy (MMD) constraint in the feature layer [19]. Constructing an adversarial game between the feature extractor and the domain discriminator by using the gradient reversal layer can force the network to learn domain-invariant features [20]. Li et al. [21] and Chen et al. [22] applied transfer learning to SAR target recognition, effectively alleviating the problem of scarce measured samples. Residual networks solve the gradient degradation problem of deep networks through skip connections [23], the channel attention mechanism can adaptively enhance important feature channels [24], and conditional generative adversarial networks realize controllable image generation by introducing constraint conditions [25].

To address the above problems, this paper proposes an ISAR target recognition method combining physics-driven data augmentation guided by detection prior information with domain adversarial transfer learning. First, the mapping relationship between scattering point projection and ISAR images is established by using the target 3D point cloud and radar observation geometric priors, and high-fidelity multi-attitude augmented images are generated by combining the 2D sinc kernel function. Then, under the unsupervised transfer learning paradigm, cross-domain recognition experiments including six types of typical aircraft targets are designed to systematically compare the recognition performance of three transfer methods: model fine-tuning, deep domain confusion (DDC), and domain-adversarial neural networks (DANN). Meanwhile, t-SNE visualization is used to analyze the feature distribution alignment ability.

The rest of this paper is structured as follows. Section 2 describes our proposed method in detail, including the detection-prior-guided physics-driven data augmentation in Section 2.1, three unsupervised transfer learning approaches (model fine-tuning, deep domain confusion, and domain-adversarial neural networks) in Section 2.2, and the experimental setup in Section 2.3. Section 3 presents the experimental results and discussion, covering recognition accuracy comparisons and t-SNE feature visualization. Section 4 concludes the paper.

## 2. Methods

### 2.1. Physics-Driven Data Augmentation Method

**Figure 1:**
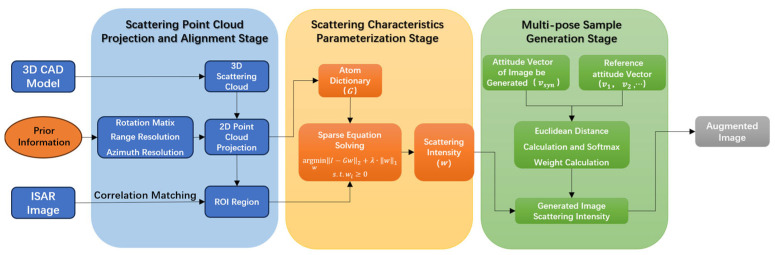
Overall Technical Framework for ISAR Image Data Augmentation.

To address the problems of scarce measured ISAR image samples and insufficient attitude coverage, a physics-driven data augmentation method guided by detection prior information is proposed in this paper. The overall technical framework of the designed data augmentation is illustrated in Figure 1. Based on a small number of ISAR images with known target categories, this method generates multi-attitude ISAR images conforming to the laws of electromagnetic scattering at the imaging mechanism level by utilizing the 3D point cloud model of targets and geometric priors of radar observation. The specific implementation steps are as follows:

First, the geometric projection matrix is constructed by using the target azimuth, roll and pitch angle information obtained from detection priors, and the 3D point cloud in actual scale is subjected to attitude transformation by using the projection matrix. Then, combined with the range and cross-range resolution parameters of the radar system, the point cloud in physical size is projected onto a discrete pixel grid. Finally, the spatial correlation between the projected image and the ISAR image is calculated, and the local sub-image of the target containing the main scattering energy is extracted from the ISAR image by the 2D cross-correlation matching algorithm.

Let the point set of the target 3D point cloud model in actual scale under the initial coordinate system be $P={\left\{P_{i}\right\}}_{i=1}^{N}$, where $P_{i}={\left[x_{i},y_{i},z_{i}\right]}^{T}$. According to the pitch angle α, roll angle β and yaw angle γ obtained from radar detection priors, the attitude transformation matrix R is constructed as:

(1) $$R=R_{yaw}(\gamma )R_{pitch}(\alpha )R_{roll}(\beta )$$

The transformed coordinates in the imaging coordinate system are given by $P_{i}^{\prime }=RP_{i}$, where $x_{i}^{\prime }$ and $y_{i}^{\prime }$ represent the cross-range and range physical positions, respectively. To ensure geometric consistency with the measured image, the physical coordinates are mapped to discrete pixel indices using the range and cross-range pixel resolutions:

(2) $$u_{i}=round\left(\frac{x_{i}^{\prime }}{∆CR}\right)$$

(3) $$v_{i}=round\left(\frac{y_{i}^{\prime }}{∆R}\right)$$

where the range pixel resolution $\Delta R=c/\left(2F_{r}\right)$ and the cross-range pixel resolutions $\Delta CR=c/\left(2f_{c}\Delta \theta \right)$ are determined by radar system parameters.

A projection density map $f_{sim}$ is constructed by accumulating scattering points at each pixel grid, i.e., $f_{sim}\left(u_{i},v_{i}\right)=f_{sim}\left(u_{i},v_{i}\right)+1$, which reflects the spatial aggregation degree of scattering points and captures the energy distribution characteristics correlated with ISAR images. Using $f_{sim}$ as the matching mask, 2D cross-correlation with the original ISAR image $f_{real}$ is computed:

(4) $$∆R\left(∆u,∆v\right)=\sum_{u,v}f_{sim}\left(u,v\right)\cdot f_{real}\left(u+∆u,v+∆v\right)$$

The offset coordinate $\left({\Delta u}^{*},{\Delta v}^{*}\right)$ with the maximum correlation determines the local sub-image $f_{sub}$ containing the main scattering energy, which after vectorization yields the observation vector I for the subsequent scattering parameter solution equation.

To parameterize the target’s electromagnetic scattering characteristics, the pixel intensity distribution in $f_{sub}$ is modeled as a sparse combination of scattering atoms. Since an ideal scattering point manifests as a point spread function (PSF) due to limited radar resolution, a 2D sinc kernel is introduced to describe the energy distribution of each scattering center:

(5) $$h_{i}\left(u,v\right)=sinc\left(\frac{u-u_{i}}{B_{r}}\right)\cdot sinc\left(\frac{v-v_{i}}{B_{c}}\right)$$

where $sinc(x)=\frac{sin(\pi x)}{\left(\pi x\right)}$, and $B_{r}$, $B_{c}$ are the broadening parameters in the range and cross-range directions determined by the radar system bandwidth and azimuth resolution.

Let the total number of scattering points be *N*, the corresponding scattering intensity coefficient vector be $w\in R^{N\times 1}$, and the total number of pixels of the extracted local sub-image $f_{sub}$ be *M*. The scattering atom $h_{i}\left(u,v\right)$ of a specific scattering point is column-wise expanded in a matrix with the same size as the local sub-image $f_{sub}$, and converted into a one-dimensional vector $g_{i}$ with a length of *M*. The vectors corresponding to all scattering points are horizontally concatenated to form an atom dictionary matrix G with a dimension of M × N:

(6) $$G=\left[g_{1},g_{2},\ldots ,g_{N}\right]$$

With the dictionary matrix G and the sub-image vector $I\in R^{M\times 1}$, the subsequent objective is to calculate the value of the intensity coefficient vector $w\in R^{N\times 1}$ corresponding to each scattering atom, such that their product Gw can approximate the measured data with the highest precision.

Considering that radar energy is physically non-negative and target scattering is typically spatially sparse (i.e., strong scattering points are only concentrated in a few positions), the solution of the intensity coefficient is transformed into a convex optimization problem with non-negativity and sparsity constraints:

(7) $$\underset{w}{\mathrm{argmin}}\frac{1}{2}{\left\|I-Gw\right\|}_{2}+\lambda \cdot {\left\|w\right\|}_{1},s.t.\;w_{i}\geq 0$$

where ${\left\|I-Gw\right\|}_{2}$ is the error term, which ensures the numerical consistency between the synthetic image and the ISAR image; ${\left\|w\right\|}_{1}$ is the $L_{1}$ norm penalty term, which is used to screen out the main scattering centers that contribute the most to imaging and eliminate redundant components; λ is the regularization coefficient, which balances the accuracy of image fitting and the sparsity of parameters. The solved vector w is the result of parametric characterization.

Based on the sinc atom dictionary G and scattering intensity coefficient w derived above, the reconstructed image vector Gw is obtained. To minimize the residual between the reconstructed image and the original target subimage, a two-dimensional search is carried out for the broadening parameters $B_{r}$ and $B_{c}$ of the 2D sinc function, so as to obtain their optimal values ${B_{r}}^{*}$ and ${B_{c}}^{*}$ at the minimum residual. The mathematical expression is given as follows:

(8) $$\left({B_{r}}^{*},{B_{c}}^{*}\right)=\underset{B_{r},B_{c}}{\mathrm{argmin}}{\left\|I-G_{B_{r},B_{c}}w_{B_{r},B_{c}}\right\|}_{F}^{2}$$

where $G_{B_{r},B_{c}}$ denotes the atom dictionary constructed with broadening parameters $B_{r}$ and $B_{c}$; $w_{B_{r},B_{c}}$ is the corresponding scattering intensity coefficient solved by Equation (7); ${\left\|\cdot \right\|}_{F}^{2}$ is the Frobenius norm, which is used to quantify the overall discrepancy between two images.

Since the original samples are typically discrete and sparse in the attitude space distribution, and the target electromagnetic scattering characteristics are highly sensitive to the observation perspective, it is imperative to establish a modeling mechanism that can characterize the dynamic evolution of scattering characteristics with the observation attitude, so as to realize the data augmentation of the ISAR image dataset in the attitude angle dimension.

The ISAR imaging characteristics of the target are jointly determined by the relative geometric relationship between the radar observation line and the target attitude. To comprehensively describe the attitude difference, a three-dimensional attitude state vector $v={\left[\alpha ,\beta ,\gamma \right]}^{T}$ of the attitude space composed of the pitch angle α, roll angle β and yaw angle γ is constructed. If there are a total of *K* reference ISAR image data with known target attitude information, for the attitude vector $v_{syn}$ of the generated image and the attitude vector $v_{k}$ of the *k-th* reference ISAR image, the weighted Euclidean distance $d_{k}$ in the attitude space is defined as:

(9) $$d_{k}=\sqrt{{\left(\alpha_{syn}-\alpha_{k}\right)}^{2}+{\left(\beta_{syn}-\beta_{k}\right)}^{2}+{\left(\gamma_{syn}-\gamma_{k}\right)}^{2}}$$

The radar scattering characteristics exhibit significant nonlinearity with the variation of the observation perspective. To ensure the normalization constraint of weights, the Softmax function is introduced to convert the Euclidean distance $d_{k}$ into the weighted coefficient $\alpha_{k}$:

(10) $$\alpha_{k}=\frac{exp\left(-d_{k}\right)}{\sum_{j=1}^{K}exp(-d_{j})}$$

The scattering intensity coefficient vector $w_{syn}$ of the generated image is obtained by weighting and summing the intensity vectors $w_{k}$ under each reference attitude using $\alpha_{k}$. It can be inferred from Equation (9) that when $v_{syn}$ is close to a certain reference attitude, the scattering intensity coefficient under this reference attitude will dominate. The calculation of the scattering intensity coefficient vector $w_{syn}$ of the generated image is given by:

(11) $$w_{syn}=\sum_{k=1}^{K}\alpha_{k}{\cdot w}_{k}$$

According to the point cloud projection method described before, the 3D point cloud set P is subjected to attitude rotation and plane projection again to obtain the two-dimensional physical coordinate set ${\left\{\left(x_{i}^{*},y_{i}^{*}\right)\right\}}_{i=1}^{N}$ under the new attitude. Subsequently, combined with the range pixel resolution ∆R and cross-range pixel resolution ∆CR of the radar system, the discrete pixel indices ${\left\{\left(u_{i}^{*},v_{i}^{*}\right)\right\}}_{i=1}^{N}$ of each scattering point in the new image matrix are calculated. Then, according to the method described before, the 2D sinc kernel function atoms $h_{i}^{*}(u,v)$ and the atom dictionary matrix $G^{*}\in R^{N\;\times N}$ are constructed from the discrete pixel indices ${\left\{\left(u_{i}^{*},v_{i}^{*}\right)\right\}}_{i=1}^{N}$. Combined with the aforementioned theory, the fused scattering intensity coefficient vector $w_{syn}$ is obtained, and the generated image vector $I_{syn}\in R^{M\times 1}$ is further derived as:

(12) $$I_{syn}=G^{*}w_{syn}$$

Based on the above steps—pose transformation, pixel mapping, construction of the sinc kernel atom dictionary, and fusion of scattering coefficients—an ISAR simulated image that meets the pose requirements of the target domain is finally generated. This process fully utilizes the detection prior information and achieves a physically consistent transformation from the 3D point cloud to the 2D image, thereby providing high-fidelity augmented samples for subsequent cross-domain recognition.

### 2.2. Unsupervised Domain Transfer Learning Recognition Method

In the inverse synthetic aperture radar (ISAR) target recognition task, the performance of deep learning models is highly dependent on the scale and diversity of labeled samples. Although an ISAR augmented dataset is constructed in this paper via physical modeling and statistical alignment methods, which effectively compensates for perspective gaps and initially reduces distribution discrepancies, practical applications are subject to variations in radar system parameters and the effects of complex electromagnetic environments, leading to persistent distribution differences between generated and measured samples—namely, the domain gap issue between augmented data and measured samples. To address this problem, this section proposes the adoption of transfer learning techniques, where the augmented dataset generated through parametric modeling is designated as the labeled source domain and the simulated dataset to be recognized as the unlabeled target domain. By mining knowledge from the source domain to assist the target domain recognition task, research on cross-domain knowledge transfer and recognition under the unsupervised setting of the target domain is conducted. Simulation experiments are designed to systematically compare the cross-domain recognition performance of three transfer learning methods—model fine-tuning, deep domain confusion (DDC), and domain-adversarial neural networks (DANN)—on the dataset constructed by the data augmentation method proposed in this paper.

#### 2.2.1. Model Fine-Tuning

Model fine-tuning is the most fundamental method in transfer learning. Since the target domain dataset is set to have no category labels, the model cannot perform any form of supervised learning on the target domain. In unsupervised domain adaptation (UDA) tasks, model fine-tuning is generally employed as the baseline for performance evaluation. Owing to the significant distribution difference between radar images and ImageNet natural images, in this subsection, the shallow parameters (Layer 1, Layer 2) of ResNet-18 are frozen, and ResNet-18 is utilized as the feature extractor. A feature classifier is constructed based on the fully connected layer according to the actual task requirements, and only the deep network and the classifier are fine-tuned during the training process. This method is adopted as the baseline scheme of the experiment to verify the cross-domain recognition performance without any distribution alignment processing.

#### 2.2.2. Deep Domain Confusion

Deep domain confusion (DDC) is one of the representative frameworks that first introduced domain adaptation theory into deep convolutional neural networks. It is improved on the basis of the traditional CNN structure, and realizes explicit feature alignment by introducing an adaptation layer and a joint loss function. The adaptive method (metric criterion) adopted in this paper is the widely used MMD criterion. The loss function of the DDC method is expressed as:

(13) $$l=l_{c}\left(D_{s},y_{s}\right)+\lambda l_{MMD}(D_{s},D_{t})$$

where l is the total loss (or joint loss); $l_{c}\left(D_{s},y_{s}\right)$ is the cross-entropy classification loss of the labeled augmented data in the source domain; $l_{MMD}(D_{s},D_{t})$ is the transfer loss for quantifying the feature distribution discrepancy between the two domains; λ is the balance factor, which is used to regulate the importance of feature distribution alignment in the model optimization process.

**Figure 2:**
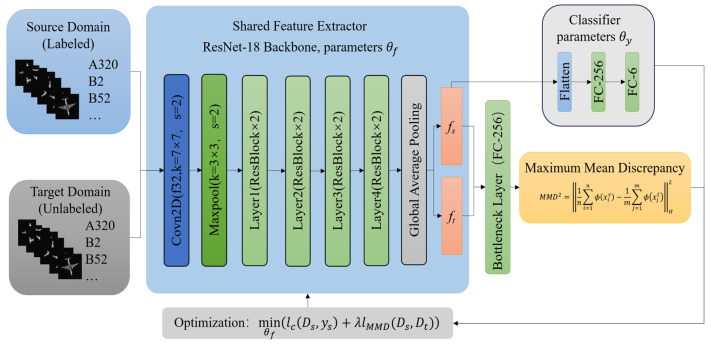
Architecture of the deep domain confusion (DDC) model used in this paper. The model consists of a ResNet-18 feature extractor, a bottleneck layer for dimension reduction, and a maximum mean discrepancy (MMD) module for explicit feature distribution alignment between the labeled source domain and unlabeled target domain.

Referring to the DDC architecture, an adaptive metric layer is introduced after the global average pooling layer of ResNet-18 in this paper. As shown in Figure 2, after the features from the source and target domains pass through the feature extractor, they first enter the bottleneck layer for dimensionality reduction mapping, reducing the 512-dimensional features output by ResNet-18 to 256 dimensions. Then, the MMD distance (adaptive loss) between the dimensionality-reduced features of the two domains is calculated, and combined with the classification loss as the joint optimization objective. In the backpropagation training process, this mechanism compels the model to realize the distribution alignment of the source and target domains in the feature space by explicitly reducing the feature distribution discrepancy between the two domains, thereby enhancing the generalization recognition capability of the model for the target domain simulated data.

#### 2.2.3. Domain-Adversarial Neural Networks

**Figure 3:**
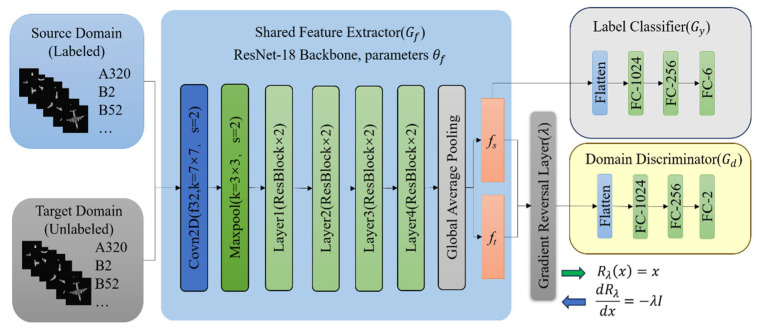
Architecture of the domain-adversarial neural network (DANN) model adopted in this paper. It includes a shared ResNet-18 feature extractor, a label classifier for target recognition, and a domain discriminator connected via a gradient reversal layer (GRL) to achieve domain-invariant feature learning.

Inspired by the “generator-discriminator” game paradigm in generative adversarial networks (GANs), domain-adversarial neural networks (DANN) are introduced in this subsection to address the feature shift problem in cross-domain recognition. DANN is a technology that renders the features learned by the neural network indistinguishable from their original data domains. It draws on the adversarial training concept of GANs, and its objective is to learn domain-invariant features that are universally applicable to both the source and target domains. The DANN structure typically consists of three components: a feature extractor $G_{f}$, a label predictor $G_{y}$, and a domain discriminator $G_{d}$. In this paper, the feature extractor $G_{f}$ is based on ResNet-18 and is responsible for extracting high-dimensional features from the input images. In the experiment, the classification head of the original network is removed, and the feature output after the global average pooling layer is retained, with a feature dimension of 512. The label predictor $G_{y}$ is composed of a multi-layer fully connected network, including two hidden layers (with dimensions of 1024 and 512 respectively) and a ReLU activation function. Overfitting is suppressed by Dropout (with a ratio of 0.5), and the output layer is mapped to 6 categories to adapt to the target recognition task in this paper. The domain discriminator $G_{d}$ is connected to the feature extractor through a gradient reversal layer. This module is composed of a three-layer fully connected structure, with hidden layer dimensions of 1024 and 256 respectively. LeakyReLU is selected as the activation function to maintain gradient activity, and the output dimension is 2, which is used to discriminate whether the features are derived from the source domain augmented data or the target domain simulated samples. Its structure is illustrated in Figure 3.

In the domain-adversarial neural network, the gradient reversal layer (GRL) serves as the core component enabling domain adversarial learning. It acts as an identity mapping in forward propagation, and the gradient is multiplied by a negative inversion coefficient $-\lambda_{p}$ in backpropagation. To ensure the stability of adversarial training, the model introduces a gradient reversal mechanism dynamically adjusted according to the training process in the GRL. In the backpropagation stage, the GRL receives the gradient from the domain discriminator, realizes the reverse transformation of the gradient by multiplying it by the negative inversion coefficient $-\lambda_{p}$ and transmits it back to the feature extractor.

(14) $$\lambda_{p}=\frac{2}{1+exp\left(-\gamma \cdot p\right)}-1$$

where p=epoch/totalˍepoch, representing the current training progress; the hyperparameter γ is a scaling factor that controls the growth rate of the inversion coefficient $\lambda_{p}$. When γ is small, $\lambda_{p}$ grows relatively gently; when γ is large, the variation curve of $\lambda_{p}$ with epoch rises rapidly at a small p, which implies that the adversarial mechanism is strongly involved in the early stage of training. In this experiment, the hyperparameter γ is set to 8.

The core logic of this dynamic adjustment strategy is as follows: in the early stage of training, the value of p is small and $\lambda_{p}$ approaches 0. At this time, the model is dominated by the classification loss, which ensures that the feature extractor first establishes the basic characterization capability for the source domain samples, laying a stable feature foundation for the subsequent adversarial game. In the middle stage of training, $\lambda_{p}$ exhibits a moderate nonlinear growth, which avoids the damage to the stability of the feature extractor caused by the sharp increase of gradient reversal intensity, and realizes a smooth transition from classification task-oriented learning to domain alignment-oriented learning. In the late stage of training, $\lambda_{p}$ gradually approaches 1, and the enhanced reverse gradient compels the feature extractor to extract domain-invariant features belonging to both the source and target domains.

### 2.3. Experimental Setup

**Figure 4:**
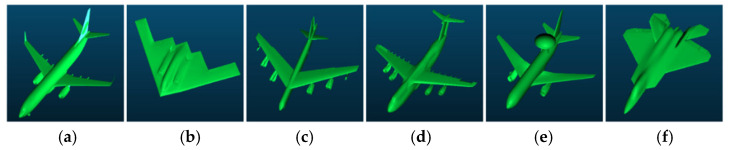
3D CAD model examples of six aircraft target categories: (**a**) A, (**b**) B2, (**c**) B52, (**d**) C, (**e**) E, (**f**) F.

**Figure 5:**
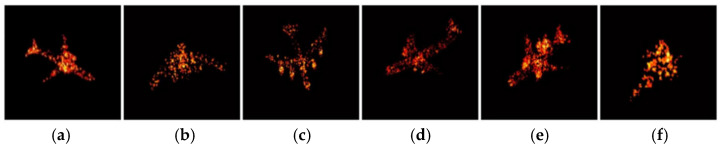
ISAR simulation image examples of six aircraft target categories: (**a**) A, (**b**) B2, (**c**) B52, (**d**) C, (**e**) E, (**f**) F.

The experiments in this section aim to verify the effectiveness of the data augmentation method proposed in Section 2.1 in improving the ISAR image recognition capability under the transfer learning framework. To this end, a cross-domain recognition dataset composed of a source domain constructed by the augmented dataset and a target domain constructed by simulated ISAR images is established. It should be noted that there are currently no publicly available measured ISAR image datasets for deep learning research; therefore, both the source and target domains are constructed using simulated data. The specific construction steps of the source and target domain datasets are as follows:

(1) Six representative aircraft targets are selected in the experiment, including Airbus A320, B-2 bomber, B-52 bomber, C-160 transport aircraft, E-767 early warning aircraft and F22 fighter jet, whose labels in the classification task are defined as [‘A’,’B2’, ‘B52’, ‘C’, ‘E’, ‘F’]. The 3D CAD model examples and ISAR simulation image examples of these six aircraft target categories are displayed in (a), (b), (c), (d), (e), (f) of Figure 4 and Figure 5 respectively. These six target categories exhibit significant differences in fuselage scale and structural characteristics, which can effectively evaluate the discriminability of the model for multi-category targets.
(2) 3D CAD modeling is performed on the six aircraft target categories, and ISAR imaging of the aircraft targets is carried out by using the physical optics method in the FEKO 14.0 electromagnetic simulation software. The radar simulation parameters are presented in Table 1. In the simulation experiment, the transmitted waveform bandwidth is 300 MHz, the range resolution is 0.5 m, and the azimuth resolution corresponding to a center frequency of 10 GHz and a synthetic aperture cumulative rotation angle of 3° is 0.287 m. Five simulated ISAR images obtained under five different attitudes of each target relative to the imaging plane are employed as reference images containing category information and prior information for each target category. After obtaining the reference images of each target category, point cloud sampling of the targets is performed by using CloudCompare v2.11.3 software, and the ISAR image augmentation method proposed in this paper is used to generate multi-attitude samples. The attitude selection scheme for each target category is as follows: the pitch angle is set to 10°; the yaw angle is generated from 0° to 360° at an interval of 2°; the roll angle is generated from 20° to 60° at an interval of 10°. Therefore, the size of the data augmentation set for each target category is 900 images, and a total of 5400 augmented data images are generated. This dataset is used as the source domain dataset with label information for transfer learning.
(3) ISAR imaging of the six aircraft target categories is carried out by using the physical optics method in the FEKO electromagnetic simulation software. The attitude selection scheme for each target category is as follows: the pitch angle is set to 10°; the yaw angle is generated from 0° to 360° at an interval of 5°; the roll angle is generated from 30° to 50° at an interval of 10°. Therefore, 216 simulated ISAR images are generated for each target category. To ensure the quality and feature descriptiveness of the unlabeled data, samples with excessive specular reflection from local structures that obscures the target contour and weakens feature descriptiveness are manually excluded, retaining 200 images per category, resulting in a total of 1200 images. This curated dataset is used as the target domain dataset without label information for transfer learning.

**Table 1:** Simulated radar parameters.

Parameter	Value
Carrier frequency	10 GHz
Signal bandwidth	300 MHz
Sampling rate	450 MHz
Pulse repetition frequency (PRF)	200 Hz
Pulse width	8 μs
Synthetic aperture angle	3°
Range sampling points	12,800
Cross-range sampling points	690

All images are uniformly resized to 224 × 224 pixels, and ResNet-18 is adopted as the backbone network. The Adam optimizer with an initial learning rate of 0.0001 is used for the model fine-tuning method; the SGD optimizer with a balance factor λ = 5 is employed for the DDC method; the Adam optimizer with a dynamic inversion coefficient hyperparameter γ = 8 is utilized for the DANN method. Finally, t-SNE dimensionality reduction visualization is adopted to analyze the feature distribution alignment effect extracted by each method, and the cross-domain recognition performance of the three transfer learning methods is comprehensively evaluated.

## 3. Results and Discussion

The recognition accuracies of the three transfer learning methods on the target domain are presented in Table 2. The baseline fine-tuning approach yields only 68.92% recognition accuracy on the target domain, confirming the presence of significant domain shift between the physics-augmented source domain and the simulated target domain. By introducing the maximum mean discrepancy (MMD) loss for explicit feature distribution alignment, the DDC method improves the accuracy to 85.75%, demonstrating the necessity of domain adaptation for cross-scenario recognition. In contrast, the adopted DANN model incorporates a dynamic gradient inversion coefficient in the adversarial training process and achieves 99.5% recognition accuracy, which proves that the model learns domain-invariant features.

**Figure 6:**
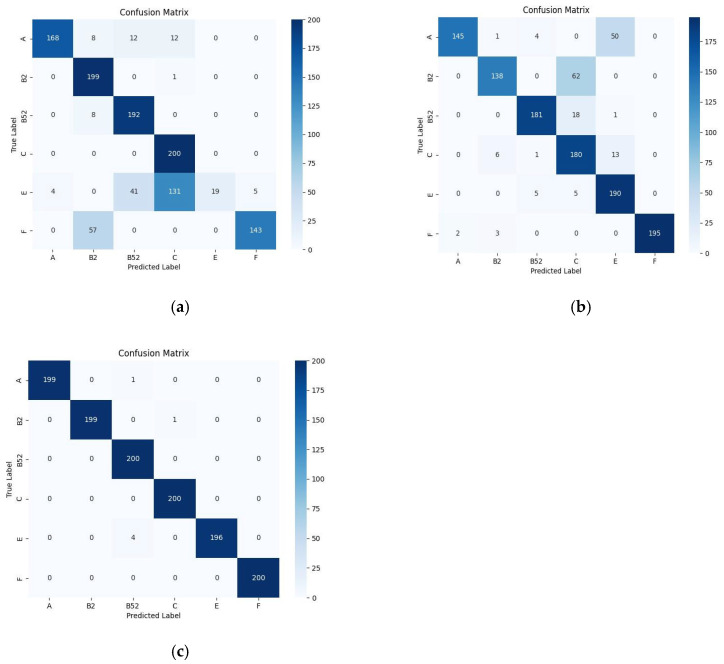
Confusion matrices of three transfer learning methods on the target domain dataset: (**a**) model fine-tuning, (**b**) deep domain confusion (DDC), and (**c**) domain-adversarial neural network (DANN). The results show that DANN achieves nearly perfect classification with all diagonal elements close to 1.0, indicating the best cross-domain recognition performance.

As visualized in the confusion matrices of Figure 6, the fine-tuning baseline produces severe inter-class confusions, particularly between ‘E’ and ‘C’, as well as between ‘A’ and ‘B52’. Although DDC alleviates partial misclassification errors, indistinct category boundaries still persist in its prediction results. Benefiting from adversarial domain-invariant feature learning, the DANN model realizes error-free classification for all six aircraft categories, with diagonal values approaching 1.0. These results verify that the DANN framework effectively eliminates domain discrepancy while preserving strong inter-class discriminability.

**Figure 7:**
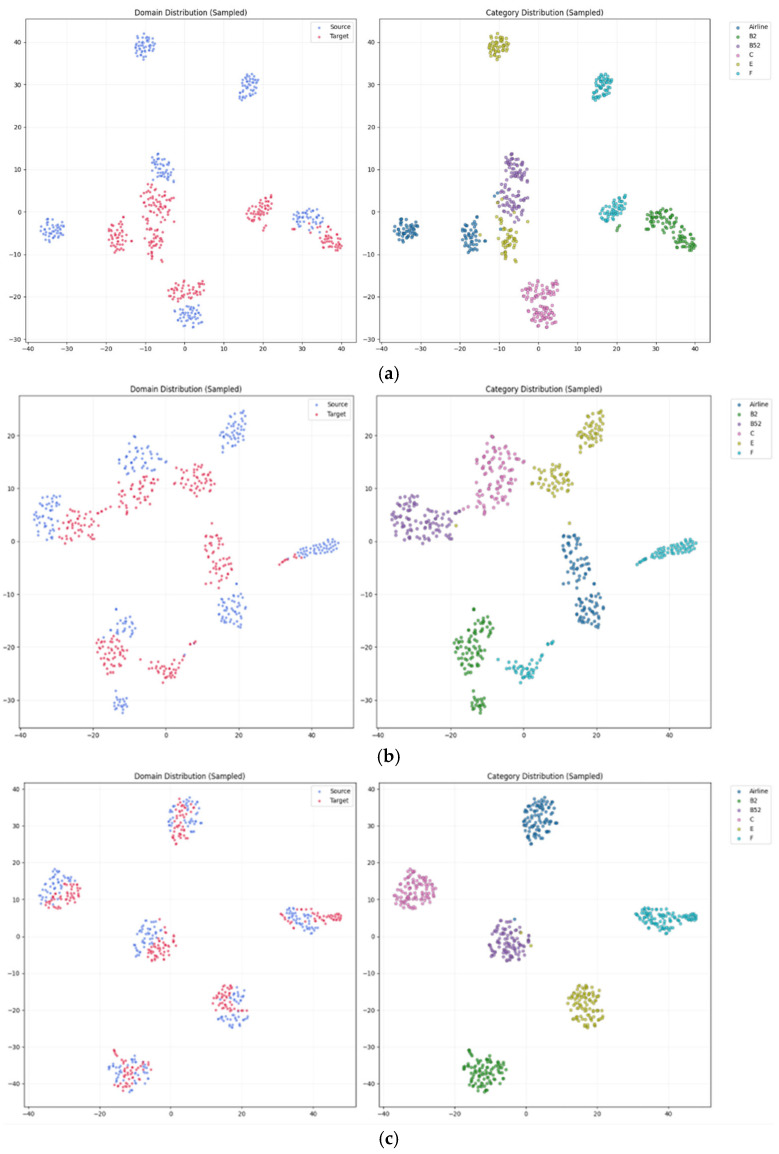
t-SNE visualization of feature distributions extracted by three transfer learning methods: (**a**) model fine-tuning, (**b**) deep domain confusion (DDC), and (**c**) domain-adversarial neural network (DANN). For each subfigure, the left panel shows domain alignment (source vs. target), and the right panel shows inter-class discriminability. DANN achieves the most overlapping domain distribution and the clearest inter-class clustering.

To further elucidate the effects of different transfer learning methods on feature distribution alignment, the t-SNE nonlinear dimensionality reduction algorithm is adopted to map high-dimensional features onto a 2D plane for visual analysis. Figure 7 presents the visualization results of the feature distributions extracted by the three methods, with the left subplot colored by domain (source domain and target domain) and the right subplot colored by target category.

Figure 7a shows that the features from the source and target domains extracted by the model fine-tuning method exhibit an obvious separation state in the two-dimensional space, with a large distance between the centroids of the two domains and a significant domain isolation phenomenon. This indicates that supervised training only on the source domain cannot eliminate the domain shift. Figure 7b reveals that the DDC method realizes the initial fusion of source and target domain features through the MMD constraint, and the overlapping degree of sample points from the two domains is increased, yet the problem of fuzzy category boundaries still persists. Some target domain samples fall into the incorrect category regions, which accounts for the low recognition accuracy of certain categories. Figure 7c demonstrates that the features extracted by the DANN method exhibit a deep overlapping distribution of source and target domains in the two-dimensional space, and the six target categories form clustering clusters with clear boundaries and no mutual overlap. This result proves that the DANN model maintains strong inter-class discriminability while eliminating the domain shift, thus achieving a high recognition rate of 99.5%.

## 4. Conclusions

This paper presents an ISAR target recognition scheme for spaceborne radar combining detection prior-guided physics-driven data augmentation and domain-adversarial transfer learning. Based on 3D point clouds and radar observation geometry, it constructs the projection mapping between scattering points and ISAR images, and generates high-fidelity multi-attitude ISAR images via 2D sinc kernel energy distribution modeling, effectively alleviating the shortage of measured ISAR samples. Comparing three unsupervised transfer learning methods (model fine-tuning, DDC, DANN), the effectiveness of the proposed augmentation method is verified, with the improved DANN with dynamic gradient inversion coefficient achieving 99.5% cross-domain recognition accuracy under ideal simulation conditions while maintaining strong inter-class discriminability. It should be clarified that the conclusions are derived from controlled simulation environments, and their generalization and robustness are still constrained by multiple factors: the quantitative influence mechanism of signal-to-noise ratio (SNR) on scattering feature extraction and sinc kernel parameter fitting, the optimization of target–clutter separation strategies in complex clutter (ground/sea clutter) scenarios, the robustness compensation methods for incomplete prior information (3D point cloud accuracy, attitude angle measurement errors), and the impact of target local structure changes and component occlusion on the fidelity of augmented samples, all of which require further quantitative analysis and verification in subsequent studies. In summary, the proposed framework exhibits physical interpretability and few-shot adaptability in theory; future work will need to conduct multi-scenario comparative experiments with measured data, deeply explore the coupling mechanism of the aforementioned factors, and provide more rigorous technical support for engineering applications.

## Figures and Tables

**Table 2:** Recognition accuracies of different transfer learning methods on the target domain.

Method	Target Domain Recognition Accuracy
Model fine-tuning	68.92%
Deep domain confusion	85.75%
Domain-adversarial neural networks	99.5%

## Data Availability

The original contributions presented in this study are included in the article. Further inquiries can be directed to the corresponding authors.
